# Supplementary material for: Design of defect spins in piezoelectric aluminum nitride for solid-state hybrid quantum technologies
Source: Sci Rep. 2016 Feb 15;6:20803. doi: 10.1038/srep20803 (PMC4753507; doi:10.1038/srep20803)
Supplement: Supplementary Information [file srep20803-s1.pdf]

Supplementary Information for "Design of defect spins in piezoelectric aluminum nitride for solid-state hybrid quantum technologies"

Hosung Seo[1,*], Marco Govoni[1,2], and Giulia Galli[1,2]

[1]The Institute for Molecular Engineering, The University of Chicago, Chicago, IL, USA

[2]Materials Science Division, Argonne National Laboratory, Argonne, IL, USA

1. Error estimation for the singlet-triplet energy gap (total energy differences) and the defect level positions in the band gap (eigenvalue differences)

In any *ab-initio* calculations, there are two types of errors: numerical and theoretical errors. The latter ones stem from the level of theory chosen to describe the system, either density functional theory (DFT) or a higher level of theory (e.g. many body perturbation theory); within DFT, theoretical accuracy is determined by the chosen exchange and correlation functional. For a given choice of theory, numerical errors originate from the basis sets and pseudopotentials used, the size of the unit cell and k-point sampling and possibly from additional numerical parameters when carrying out, e.g. GW calculations. The accuracy of computed eigenvalue differences and total energy differences is affected in a different way by the chosen theoretical and numerical approximations. For example, it is well known that when using DFT, even local exchange correlation functionals may yield rather accurate results for *total energy differences* (and hence structural properties of materials), but they cannot reproduce accurately measured energy gaps (*single particle energy differences*).

In the following we discuss about the theoretical and numerical errors in our DFT calculations. We drew our main conclusions from *total energy differences* between singlet and triplet configurations and from *eigenvalue differences*. In our paper we report that: (1) the triplet state of the V_N^- defect is more stable than the singlet state under realistic strain conditions: by 250 meV (*total energy difference*) under 3% compressive uniaxial strain, and by 80 meV (*total*

* Email: hseo@uchicago.edu

energy difference) under 4% tensile biaxial strain. (2) The single particle wave-functions associated with the triplet spin state of V_N^- are highly localized in the band gap of AlN: these states are about 2.2 eV and 1.7 eV (*eigenvalue differences*) below the conduction band minimum for 3% compressive uniaxial and 4% tensile biaxial cases, respectively. We show below that in our calculations numerical errors associated to total energy differences are 0.01 eV (or 0.02 eV at most) and those associated with eigenvalue differences are about 0.2 eV. Hence our conclusions are robust.

Numerical errors: We checked the numerical errors in our defect calculations at the DFT-PBE level (that is, at fixed level of theory) within the periodic boundary condition.

(1) *Numerical errors on absolute total energy values of AlN introduced by k-point sampling and plane-wave cutoff energy*: We used a 2×2×2 k-point mesh for the 480-atom supercell calculations, corresponding to a 10×10×8 k-point mesh for the 4-atom primitive unit-cell of aluminum nitride. With this k-point sampling, we achieved convergence for the *absolute value* of total energies within1.5×10^{-6} eV per atom. We also used a 2×2×2 k-point mesh for a smaller 96-atom supercell, mainly for PBE0 calculations. This choice of k-point mesh corresponds to a 6×6×4 mesh for the 4-atom primitive unit-cell calculation, and the convergence is within 3.1×10^{-4} eV per atom. For the plane-wave cutoff energy, we used 75 Ry (=1020.4 eV), with which we obtained absolute total energy values converged within 5.0×10^{-3} eV per atom.

(2) *Numerical errors in total energy differences*: We checked the convergence of the total energy difference between two different spin states (S=0 and S=1) of V_N^- in AlN as a function of supercell size, cutoff-energy, and k-point sampling. For our supercell-size tests, we used 96-atom, 360-atom, and 480-atom supercells along with a 2×2×2 k-point mesh and 75 Ry plane-wave cutoff energy. We found that the energy difference is well-converged even for the relatively small 96-atom supercell. The difference between total energies per defect obtained with 96-atom and 480-atom cells and the same 2×2×2 k-point mesh under no strain is only 3 meV per defect. Similar results on total energy differences were obtained for a wide range of strains, as shown in Figure 2(a) and 3(a) of the paper. Then, using a 96-atom supercell, we performed convergence tests for the singlet-triplet total energy difference as a function of k-point sampling and plane-

wave cutoff energy, as shown in Figure S1. We found that the total energy difference is converged within 5 meV, when using a 2×2×2 k-point mesh along with a 75 Ry cutoff energy.

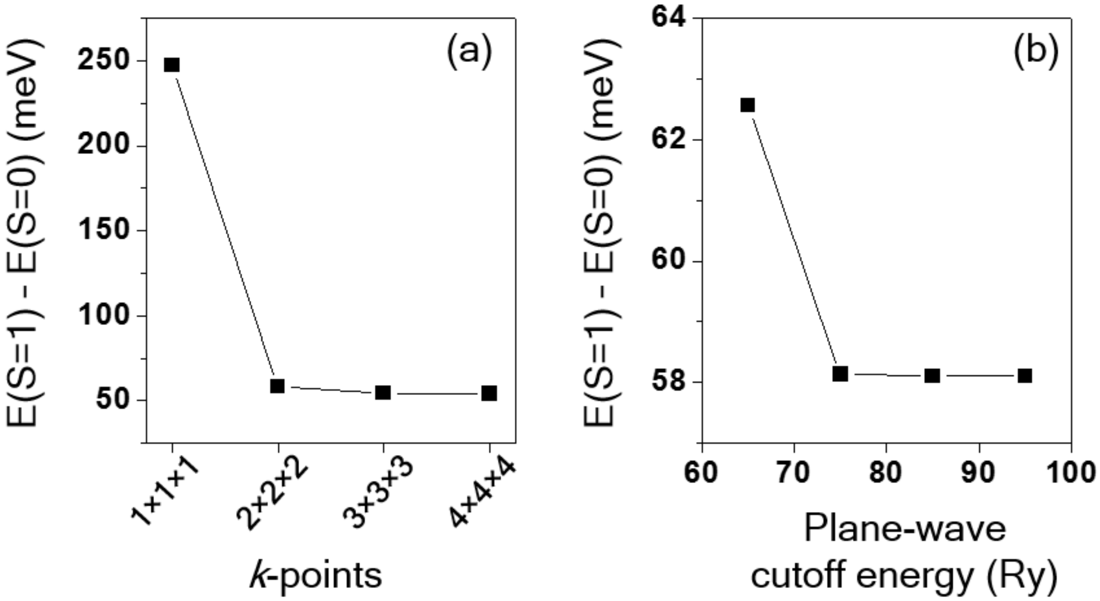

Figure S1. Total energy difference between the S=1 and S=0 state of V_N^- under no strain as a function the k-point sampling (all points obtained with plane-wave cutoff of 75 Ry) (a) and the plane-wave cutoff energy (all points obtained for a 2×2×2 k-point mesh) (b). A 96-atom supercell was used.

(3) *Numerical errors eigenvalue differences:* For a charged defect calculation, large supercells are necessary so as to minimize artificial interactions between periodic images. In general there may be two main artificial interactions due to the use of finite-size supercells: overlap between wave-functions of periodic images and long-range electrostatic interaction between charges of the defect. The error introduced by wave-functions' overlap can be estimated by the dispersion width of the defect energy level across the Brillouin zone. In our study, the dispersion width of a defect level in the band gap of AlN was calculated to be less than 10 meV in our 360-atom and 480-atom supercell calculations, which is a small value (by more than 1 order of magnitude) compared to total energy and single particle energy differences used to draw our main conclusions. The presence of spurious long-range electrostatic interactions may affect the position of a defect level in the band gap of AlN, with respect to the valence band edge, and the magnitude of the effect depends on the supercell size [S1]. By using a large 1920-atom supercell, we found that the magnitude of this error for *eigenvalue differences* ranges from 0.05 eV to 0.07

eV for a 480 atom supercell. However, even if we shifted the results obtained for the position of the defect energy level with a 480-atom cell by ~0.1 eV, our conclusions would be unaffected, since the defect levels of interest are located 2.2 eV and 1.7 eV below the conduction band minimum for the 3% compressive uniaxial and 4% tensile biaxial cases, respectively. Finally, using the 96-atom supercell, we also checked the convergence of the eigenvalue differences as a function of k-point sampling and the plane-wave cutoff energy difference, which is summarized in Figure S2. We found that the eigenvalue difference is converged to 5 meV, when a 2×2×2 k-point mesh along with a 75 Ry cutoff energy is used.

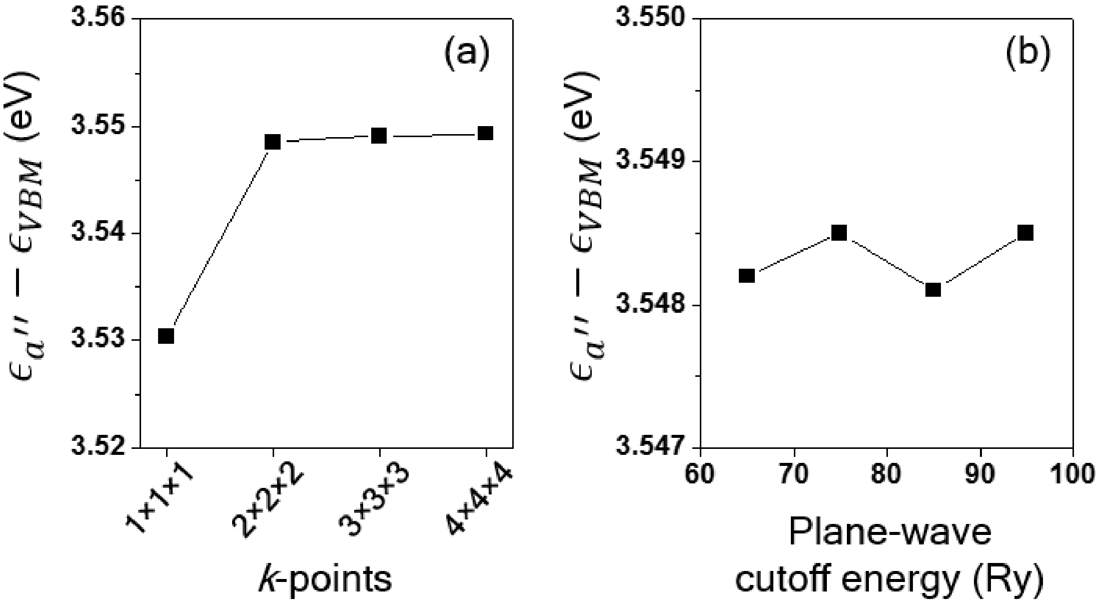

Figure S2. The position of the *a*'' defect level of the S=1 state of V_N^- under no strain with respect to the valence band edge as a function of the k-point sampling (all points obtained with plane-wave cutoff of 75 Ry) (a) and of the plane-wave cutoff energy (all points obtained for a 2×2×2 k-point mesh) (b). A 96-atom supercell is used.

<u>*Theoretical errors:*</u> These errors may stem from the level of theory chosen to draw our main conclusions.

(1) *Theoretical errors in the singlet-triplet total energy difference:* In our paper, we showed that the triplet state of V_N^- can be stabilized by straining the lattice. In order to verify if our results are robust with respect to the level of theory used, we carried out calculations with two different exchange-correlation functionals: PBE and PBE0. The rationale for the choice of PBE0 as a

higher level of theory than PBE is explained in detail in the method section. In short, a recent work of our group (Ref. [28]) showed that the electronic properties of a variety of semiconductors and insulators are best obtained using a mixing fraction of exact exchange equal to $1/\epsilon_\infty$ (where ϵ_∞ is the high frequency dielectric constant of the system). For AlN, the self-consistent value of ϵ_∞ is arount 4 (Ref. [28]), making the optimal mixing fraction equal to ~0.25, which is exactly the fraction used in the definition of the PBE0 functional. Additionally, we note that PBE0 is known to significantly reduce the delocalization error found with PBE, thus improving results on eigenvalue and total energy differences. We found that the computations based on both levels of theory produced consistent results and thus proved the robustness of our main conclusions against theoretical errors.

(2) *Theoretical errors in single particle eigenvalue differences:* Although total energy differences can be described reasonably well using the semi-local PBE functional, it is well known that PBE severely underestimates the band gap (eigenvalue difference) of many semiconductors and insulators, including AlN. Thus, PBE is not expected to be a reliable way of calculating defect level positions in the band gap. Hence we used many body perturbation theory, in particular G_0W_0@PBE calculations. For the band gap of *w*-AlN, we obtained 5.94 eV within the G_0W_0@PBE approximation using our 480-atom supercell with a point defect, which is in very good agreement with the previous G_0W_0@LDA results of 5.8 eV [S2] and 6.08 eV [S3] obtained for the pristine bulk. We also carried out calculations using the PBE0 functional with the same 480-atom supercell structure (see Figure S3 and S4). By comparing the occupied defect level positions with respect to the valence band edge at the PBE0 and G_0W_0@PBE level of theories, we found that the theoretical uncertainty of the defect level positions with respect to the valence band edge is ~0.2 eV. We note that this number is much smaller (by a factor of 5 to 10) than all eigenvalue energy differences used for all conclusions reported in our paper.

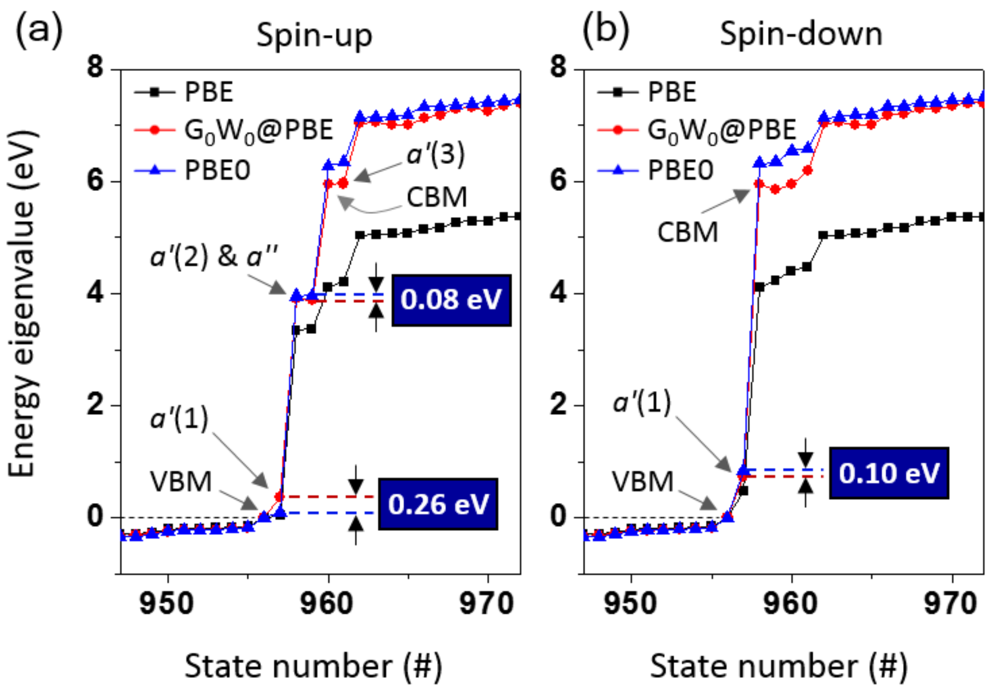

Figure S3. Positions of the bulk and defect states of the S=1 state of V_N^- in AlN calculated within PBE (black squares), G_0W_0@PBE (red circles) and PBE0 (blue triangles). Energies are referred to the valence band maxima obtained at the respective levels of theory. The spin-up and spin-down states are shown in (a) and (b), respectively.

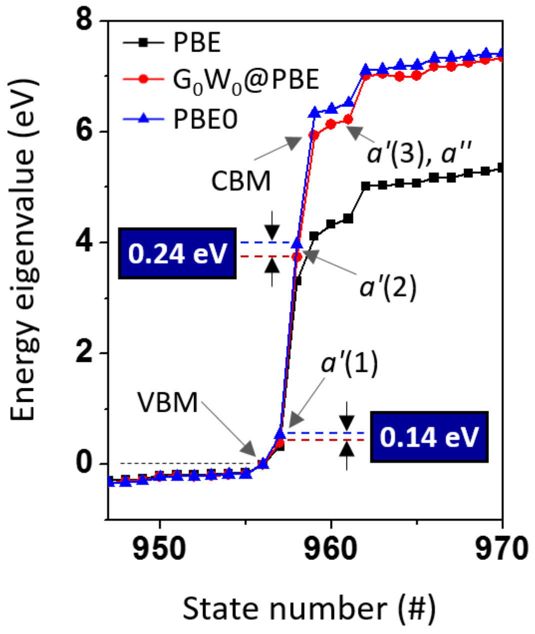

Figure S4. Positions of the bulk and defect states of the S=0 state of V_N^- in AlN calculated within PBE (black squares), G_0W_0@PBE (red circles) and PBE0 (blue triangles). Energies are referred to the valence band maxima obtained at the respective levels of theory.

2. Estimation of the finite temperature effects on the singlet-triplet stability

The temperature of AlN used in our calculations is zero, namely we only compared the total energies of the S=0 and S=1 states to determine their relative stability. At finite temperature (e.g. T=300K), we expect that vibrational contributions to the free energy would largely cancel out in the two different spin configurations, and that the relative stability of the triplet state of V_N^- compared to the singlet state, would be the same as the one determined using total energy differences. We discuss this point in detail below.

At finite T the stability of a defect is determined by the defect formation *free* energy, which can be obtained by replacing the total energies in equation (1) in the main text with the following free energies of the host (F^H) and the defect (F^D) (see Ref. [S4] for details):

$$F^H = E^H + F_{vib}^H - TS_{elec}^H, \quad \text{(S1)}$$

$$F^D = E^{D(q)} + F_{vib}^D - T(S_{config}^D + S_{elec}^D), \quad \text{(S2)}$$

where E^H and E^D are the total energy of the system without and with the defect, respectively. The electronic entropic terms (S_{elec}^H and S_{elec}^D) are negligible in our case due to the wide-gap of AlN. S_{config}^D is the configurational entropy, which is given by:

$$S_{config}^D = k_B \ln \Omega^D , \quad \text{(S3)}$$

where Ω^D is the number of different defect configurations attainable in the supercell. The vibrational free energy is given by:

$$F_{vib} = \int_0^{\infty} g(\omega)\{\frac{1}{2}\hbar\omega + k_B T \ln[1 - \exp(-\frac{\hbar\omega}{k_B T})]\}\, d\omega, \quad \text{(S3)}$$

where $g(\omega)$ is the phonon density of states. The phonon contribution to the formation free energy of any specific defect may be significant. For example, for the nitrogen vacancy center in diamond, Webber *et al.* calculated the phonon contribution to the defect formation free energy to be ranging from -0.5 eV at T=0 K to -0.86 eV at T=2000 K [S5]. We note, however, that in our work we considered the relative stability of different spin states of the same V_N^- defect, which is defined by a *difference of formation energies* (not by absolute formation energies); such difference is given by:

$$\Delta F_f = E^{D(S=1)} - E^{D(S=0)} + E_{corr}^{D(S=1)} - E_{corr}^{D(S=0)} + F_{vib}^{D(S=1)} - F_{vib}^{D(S=0)} , \quad \text{(S4)}$$

where $E_{corr}^{D(S=1\ or\ 0)}$ is a correction term for artificial electrostatic interactions present in the finite-size supercell calculations, which was computed following the work of Freysoldt, Neugebauer and Van de Walle [S4]. Note that the configurational entropic terms (S_{config}^{D}) of the S=0 and S=1 states cancel out as their defect structures have the same C_{1h} symmetry. We verified that in our calculations the difference ($E_{corr}^{D(S=1)} - E_{corr}^{D(S=0)}$) is zero. Indeed each of the energy terms is determined by the long-range electrostatic interaction between the defect charge (-*e* for both S=1 and S=0) and its periodic image, which is the same irrespective of the spin state. Therefore, the main temperature effect stems from the difference of the vibrational free energy contributions between two different spin states. Explicit first-principles calculations of the vibrational contributions are beyond the scope of our current study and we only estimated the order of magnitude based on relevant previous studies present in the literature. For the NV center in diamond, Webber *et al.* estimated the difference of the vibrational free energies of the S=1 state of NV^- and the S=1/2 state of NV^0 to be ranging from -8 meV (T=0 K) to -2 meV (T=2000 K) [S5]. Webber *et al.*, also found that the majority of phonon density of states (DOS) is determined by the host lattice and the phonon DOS of the point defect only gives rise to a very small correction (at least an order of magnitude smaller than that of the host lattice) to the host phonon DOS. Furthermore, they found that such correction is very similar regardless of the spin state of the NV center, which explains the small vibrational free energy difference between the two spin states of the NV center. Another relevant study was reported for a nitride system by Zhou *et al.* [S6]. Zhou *et al.* compared the thermodynamic stability of CrN having different magnetic states and they estimated the vibrational free energy difference between different magnetic states (e.g. ferromagnetic vs. antiferromagnetic states at T=300 K) to be about -5 meV/Cr-atom. Therefore, for our stability comparison between two different spin states of V_N^- in AlN, we expect a large cancellation of phonon contributions, similar to the case of diamond, and we estimate the error range associated with temperature effects to be 10-20 meV.

References for Supplementary Information

S1. W. Chen and A. Pasquarello, Phys. Rev B **88**, 115104 (2013).

S2. A. Rubio, J. L. Corkill, M. L. Cohen, E. L. Shirley, and S. G. Louie, Phys. Rev. B **48**, 11810 (1993).

S3. D. Cociorva, W. G. Aulbur and J. W. Wilkins, Solid State. Commun. **124**, 63-66 (2002).

S4. C. Freysoldt *et al.*, Rev. Mod. Phys. **86**, 253-305 (2014).

S5. B. T. Webber, M. C. Per, D. W. Drumm, L. C. L. Hollenberg, and S. P. Russo, Phys. Rev. B **85**, 014102 (2012).

S6. L. Zhou *et al.*, Phys. Rev. B **90**, 184102 (2014).
